# A higher baseline plasma uric acid level is an independent predictor of arterial stiffness: A community-based prospective study

**DOI:** 10.1097/MD.0000000000005957

**Published:** 2017-02-10

**Authors:** Xiao-Han Ding, Xiaona Wang, Ruihua Cao, Xu Yang, Wenkai Xiao, Yun Zhang, Yongyi Bai, Hongmei Wu, Ping Ye

**Affiliations:** a Department of Geriatric Cardiology, Chinese PLA General Hospital, Beijing; b Department of Health Care and Geriatrics, Lanzhou General Hospital of Lanzhou Military Command, Lanzhou, Gansu Province, China.

**Keywords:** arterial stiffness, community-based, longitudinal study, predictor, uric acid

## Abstract

Hyperuricemia has been demonstrated to be a risk factor for cardiovascular diseases. Though the association between uric acid (UA) and arterial stiffness has been investigated previously in patients with basic diseases, the predictive value of baseline UA level for arterial stiffness has not been conducted. We aimed at identifying the predictive role of UA for arterial stiffness prospectively.

A longitudinal follow-up study in a routine health check-up population was performed with an average follow-up of 4.8 years. The demographic information, baseline and follow-up anthropometric parameters, arterial stiffness (pulse-wave velocity, PWV), and biomarker variables including UA have been measured and analyzed.

A total of 1447 valid follow-ups were available for the final analysis. Both of the baseline and follow-up UA levels were significantly higher in the arterial stiffness groups than that in the nonarterial stiffness groups (all *P* values <0.001). The follow-up carotid-femoral PWV [(cf-PWV), *r* = 0.161, *P* < 0.001] was strongly correlated with baseline UA. At the follow-up cross-section, cf-PWV was also closely associated with UA (*r* = 0.101, *P* < 0.001). Logistic regressions revealed that a higher baseline UA level was an independent predictor of follow-up arterial stiffness assessed by cf-PWV [odds ratio (OR): 1.824; *P* = 0.046].

A higher baseline level of UA is closely related to arterial stiffness and is an independent predictor of arterial stiffening.

## Abstract 2

Supplemental Digital Content is available in the text

## 1 Introduction

Hyperuricemia has been demonstrated to be an independent risk factor for cardiovascular diseases including arterial stiffness, atherosclerosis, and hypertension.^[1–3]^ Uric acid (UA) abnormalities are also associated with hypertension, regional arterial stiffness in patients with renal failure, and diabetes mellitus (DM).^[4–8]^ Furthermore, a relationship between normal serum UA and arterial stiffness has also been reported before.^[9–11]^ UA may be involved in the pathogenesis of arterial stiffness and hypertension potentially as a result of thickening vessel walls (intima-media) by promoting smooth muscle cell proliferation and endothelial cell dysfunction.^[12–14]^

It is well known that arterial stiffness is a marker of aging including blood vessel aging, and arterial stiffness has been identified as a risk factor for prepathophysiological processes in numerous cardiovascular (such as hypertension and other cardiovascular diseases) and cerebrovascular diseases.^[15–19]^ Furthermore, there are several cardiocerebral diseases may be subsequences of arterial stiffening. Arterial stiffening is also the hallmark of normal vascular aging, and early acceleration of the stiffening process represents one of the major pathologies of the cardiovascular system.^[20]^ There are several methods for quantifying arterial mechanical properties have been proposed.^[21,22]^ The most commonly used technique is measurement of the pulse-wave velocity (PWV) which provides a noninvasive method of assessing stiffness along an arterial section in the clinical practice.^[21,23]^ Currently, PWV is used as a reproducible and valid noninvasive “gold standard indicator” in the assessment of arterial stiffness.^[23]^ Nevertheless, PWVs from different arteries usually represent stiffening in distinct regions of the vasculature. Particularly, carotid-femoral PWV (cf-PWV) and carotid-radial PWV (cr-PWV) are often applied to assess stiffness in the aorta and arterioles.

Though the previous results are inconsistent, evidence is accumulating to indicate an association between serum UA level and arterial stiffness. The previous studies have documented that the UA were associated with hypertension, arterial stiffness, and other vascular alterations.^[8,24]^ However, most of the studies have been conducted based on the patients with various disorders such as DM, hypertension, and chronic kidney disease, as well as among various ethnic groups.^[25–27]^ To the best of our knowledge, there are few reports of follow-up studies evaluating the role of the baseline level of UA in arterial stiffness. Thus, we postulated that a higher UA level may also play a critical role in aggravating arterial stiffness as evaluated by cf-PWV, and UA has a critical role in increasing arterial stiffness. We have conducted this follow-up observational study with the aim of providing novel indices for stratification and risk management of arterial stiffness.

## 2 Methods

### 2.1 Participants and procedures

A total of 1680 participants who underwent a routine health examination were recruited between September 2007 and January 2009 from the Pingguoyuan area in the Shijingshan district in this community-based follow-up cohort study. Residents who received a routine health check-up in the community were eligible for inclusion. Subjects with any of the follow conditions were excluded: infection, and neoplastic or severe liver or renal diseases.

### 2.2 Follow-up and outcome assessment

All participants were followed up for all-cause mortality, cardiovascular disease mortality, arterial stiffness, and the development of DM from the initial screening to September 2013. During a median of 4.8-year follow-up of the 1680 subjects, 181 participants were lost to follow up and were excluded from the final analysis. Thus, 1499 subjects (follow-up rate 89.2%) completed the follow-up, and 52 were excluded because of death. Finally, 1447 participants were valid for the analysis.

The present study was reviewed and approved by the Ethics Committee at the People's Liberation Army General Hospital. The study was explained in detail to all of the subjects who agreed to participate in, and all of the subjects signed informed consent forms before their examinations.

### 2.3 Clinical data collection

The participants were followed up by our trained physicians, and a standardized self-report questionnaire form was used to record demographic information, lifestyle factors, prevalent diseases, family history, and medication use. Anthropometrics were evaluated by trained medical doctors. Height was measured in centimeters using a wall-mounted measuring tape, and weight was measured using a digital scale in kilograms (without shoes). Self-reported smoking status was categorized as current, former, or never smoking. Systolic blood pressure (SBP) and diastolic blood pressure (DBP) were measured on the right arm in a sitting position after a 5-minute rest period.

### 2.4 Biomarker variable determination

Venous blood samples were obtained from subjects between 8 am and 10 am after an overnight fast (at least 12 hours). Plasma aliquots were obtained and stored at −80°C for further assays. Plasma UA, fasting blood glucose (FBG), triglyceride (TG), low-density lipoprotein cholesterol (LDL-C), and high-density lipoprotein cholesterol (HDL-C) concentrations were measured from venous blood samples using commercially available ELISA kits by Roche enzymatic assays (Roche Diagnostics GmbH, Mannheim, Germany). The concentration of plasma creatinine (Cr) was measured by an enzymatic assay (Roche Diagnostics GmbH) on a Hitachi 7600 autoanalyzer (Hitachi, Tokyo, Japan). In addition, the non-DM subjects received the standard 75-g oral glucose tolerance test.

All of the biochemical variables were measured from the blood specimens in the Clinical Laboratory Department, Chinese PLA General Hospital, following the criteria of the World Health Organization Lipid Reference Laboratories.

### 2.5 Assessment of arterial stiffness

Baseline and follow-up arterial stiffness was assessed by automatic carotid-femoral PWV (cf-PWV) measurement using a Complior SP device (Createch Industrie, Massy, France) after a 5- to 10-minute rest. PWV (m/s) = distance (m)/transit time (s). The detailed procedure was described in Appendix 1 (assessment of arterial stiffness in detail).

### 2.6 Definitions of variables

Hypertension was defined as a SBP ≥140 mm Hg and/or a DBP ≥90 mm Hg or the use of antihypertensive medications. Other variables such as body mass index (BMI) and smoking were defined in Appendix 1 (definition of variables in detail). Arterial stiffness group (AS-group) was defined as: patients with a follow-up cf-PWV ≥12 m/s.^[22,28]^ The detailed definition of variables is described in Appendix 1.

### 2.7 Statistical analysis

Normally distributed baseline continuous variables are expressed as the mean ± standard deviation (SD) and were analyzed with Student *t* tests, while the baseline dichotomous variables are presented as numbers (percentages) and compared using the Chi-square test. Nonnormally distributed variables, such as UA levels and other biomarkers, were normalized by natural logarithm transformation as necessary.

A Pearson regression analysis, a multivariable linear regression analysis, and a multicollinearity analysis were performed to evaluate the associations of UA (natural logarithm transformed) level with arterial stiffness and other parameters at both baseline and the end of the follow-up.

**Figure 1:**
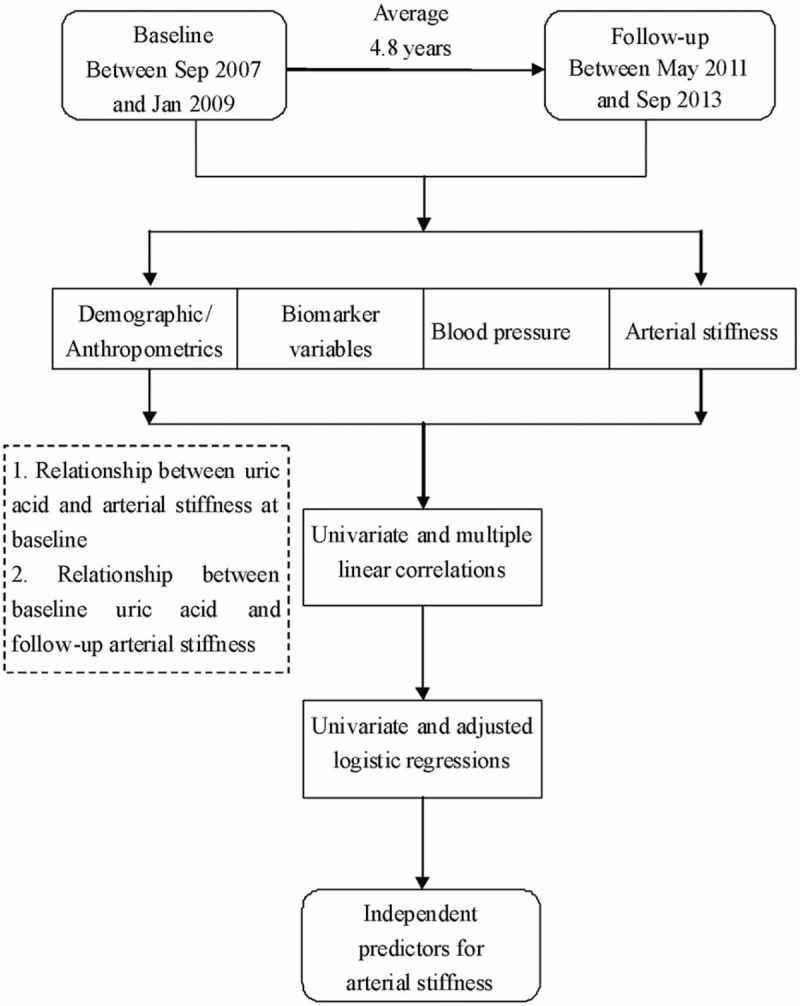
Flow diagram of this study.

Further analysis by a forward stepwise multivariable logistic regression analysis were performed to identify the association between UA (baseline) and the follow-up arterial stiffness (cf-PWV ≥12 m/s vs cf-PWV <12 m/s). Regression models were adjusted for age and sex as the independent variable (Model 1) and additionally adjusted for smoking, alcohol use (g/d), DM, SBP, DBP, TG, LDL-C, HDL-C, and Cr as the independent variables (Model 2, Fig. 1).

All analyses were performed using SPSS 19.0 for Windows (SPSS, Chicago, IL). *P*-value < 0.05 is considered to be statistically significant. Statisticians from the People's Liberation Army General Hospital were consulted regarding all of the statistical methods and results.

## 3 Results

### 3.1 Baseline clinical characteristics

The mean age of the subjects was 61.40 ± 11.4 years, and 59.98% were women. The body mass index (BMI) of the subjects in the study was 25.41 ± 3.32 kg/m^2^. Altogether, 26.26% and 18.93% of the subjects smoke and drink currently. The characteristics of the subjects categorized by UA levels at baseline are shown in Table 1.

**Table 1:**
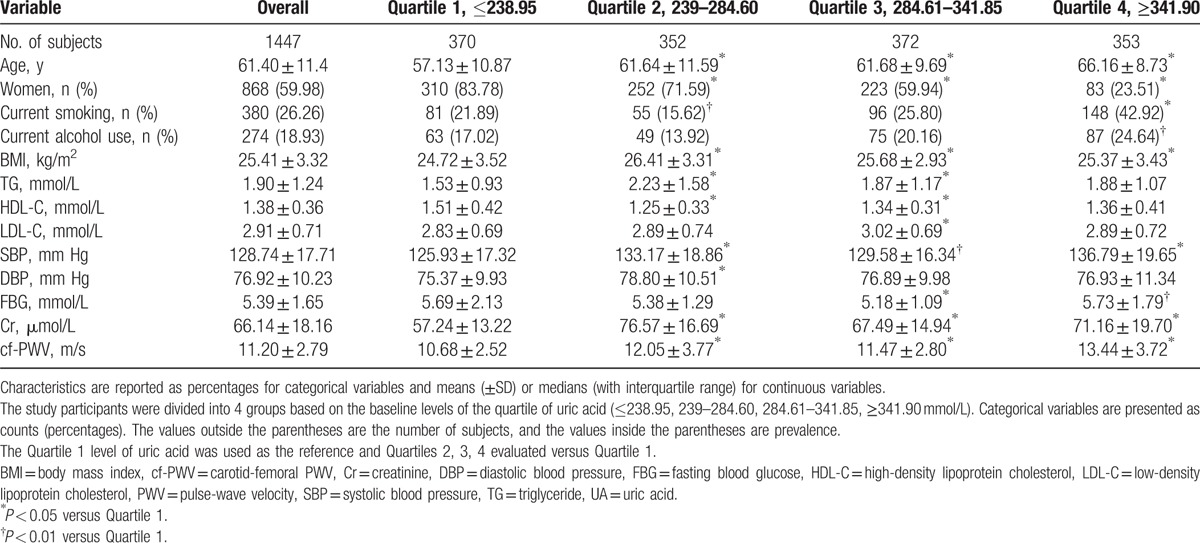
Characteristics of the subjects categorized by uric acid levels at baseline.

### 3.2 Associations between UA level and arterial stiffness at baseline

At baseline, Pearson analysis showed that the UA level was strongly related to baseline cf-PWV (*r* = 0.183, *P* < 0.001). Additionally, age (*r* = 0.509, *P* < 0.001), SBP (*r* = 0.351, *P* < 0.001), LDL-C (*r* = 0.069, *P* = 0.014), HDL-C (*r* = −0.101, *P* < 0.001), Cr (*r* = 0.140, *P* < 0.001), and TG (*r* = 0.111, *P* < 0.001) were also closely associated with cf-PWV in the univariate linear analysis (Table 1A in Appendix 2). However, in the multivariable analysis, UA level was not correlated with cf-PWV (β = 0.555, *P* = 0.065), which was adjusted by age, DBP, Cr, and TG in the multivariable linear analysis (Appendix 2, Table 1A).

### 3.3 Baseline UA level was closely associated with follow-up arterial stiffness

The UA level at baseline in the AS-group was significantly higher than that in the non-AS-group, while it was similar between the 2 groups at the follow-up cross-section (Table 2). Differences in other baseline parameters including age, BMI, cholesterol, and blood pressure between the 2 groups are shown in Table 2.

**Table 2:**
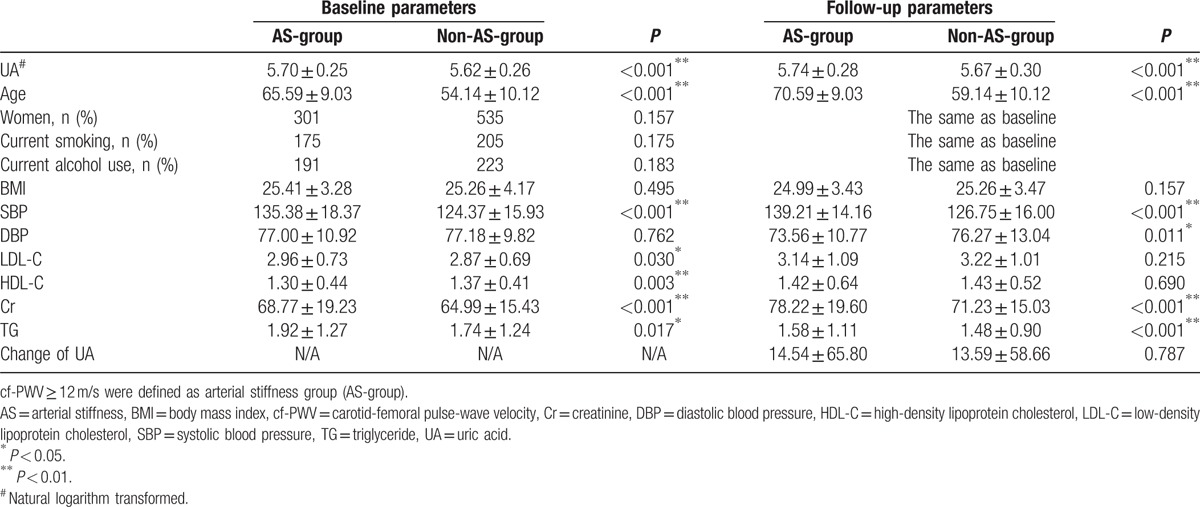
Differences in baseline and follow-up perimeters between AS and non-AS-groups.

To identify the association between baseline UA and follow-up arterial stiffness, Pearson correlation analyses were employed. The univariate analysis revealed that baseline UA (*r* = 0.161, *P* < 0.001), age (*r* = 0.533, *P* < 0.001), SBP (*r* = 0.333, *P* < 0.001), Cr (*r* = 0.176, *P* < 0.001), and TG (*r* = 0.089, *P* < 0.001) were positively correlated with the follow-up cf-PWV, while HDL-C (*r* = −0.071, *P* = 0.011) was negatively associated with the follow-up cf-PWV. Though the multiple linear regression analysis revealed no association between baseline UA and follow-up cf-PWV (β = 0.143, *P* = 0.645), the baseline age, SBP, DBP, Cr, and TG were still strongly correlated with the follow-up cf-PWV (Table 3).

**Table 3:**
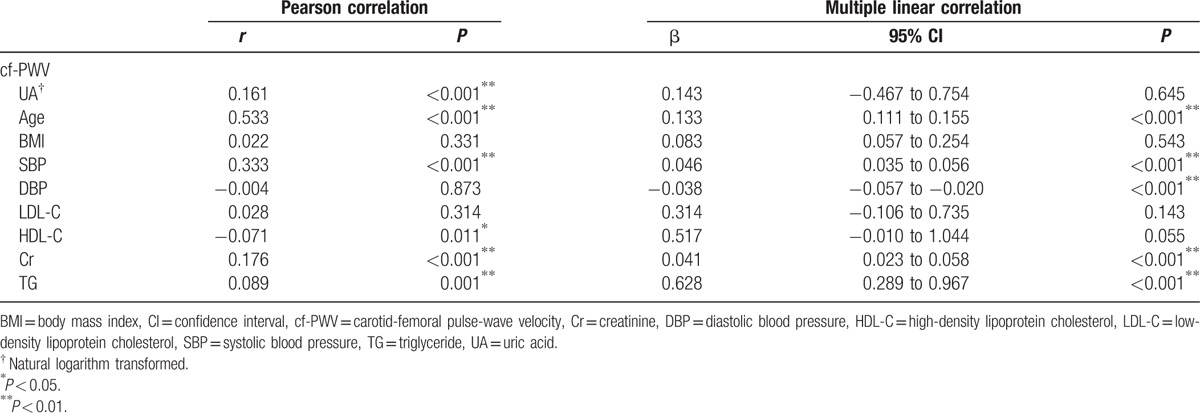
Univariate and multiple linear correlation analysis of baseline parameters and follow-up cf-PWV.

The results of the univariate and multiple Pearson linear regression analyses for cf-PWV and other parameters at follow-up cross-section are summarized in Table 4 which also showed the association between cf-PWV and UA level at the follow-up cross-section. We have also analyzed the association between the changes of UA levels and cf-PWV. However, we have observed no significant associations (Tables 2 and 4).

**Table 4:**
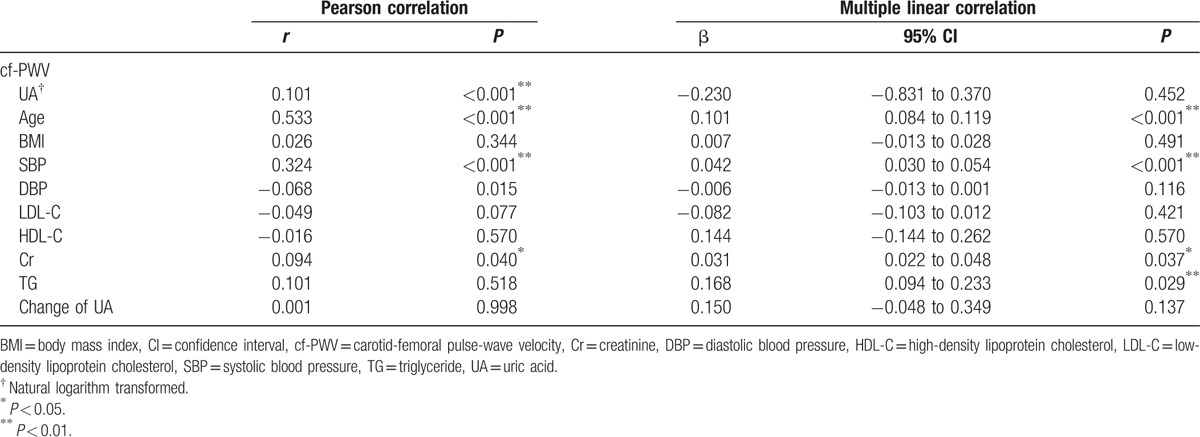
Univariate and multiple linear correlation analysis of the parameters and arterial stiffness at follow-up.

### 3.4 Logistic regressions for UA and arterial stiffness

Univariate logistic regression showed that a higher baseline UA level was a risk factor for follow-up arterial stiffness measured by cf-PWV [β = 1.263, OR (odds ratio): 3.536, 95% CI (confidence interval): 2.267–5.515, *P* < 0.001]. Additionally, a higher baseline age, SBP, LDL-C, Cr, and TG were also shown to be risk factors for arterial stiffness (all *P* values < 0.05). Whilst, a higher baseline HDL-C level was a protective factor for arterial stiffness. Furthermore, the age- and gender-adjusted model also revealed that a higher baseline UA level was associated with a greater risk for arterial stiffness (β = 0.689, OR: 1.991, 95% CI: 1.123–3.531, *P* = 0.018). Importantly, a higher baseline UA level remained a predictor of arterial stiffening measured by cf-PWV at follow-up (β = 0.601, OR: 1.824, 95% CI: 0.960–3.465, *P* = 0.046) in Model 2, which was adjusted by age, DBP, SBP, and levels of TG, HDL-C, LDL-C, and Cr. Thus, these results demonstrate that a higher baseline UA level is an independent predictor of central arterial stiffening (Table 5).

**Table 5:**
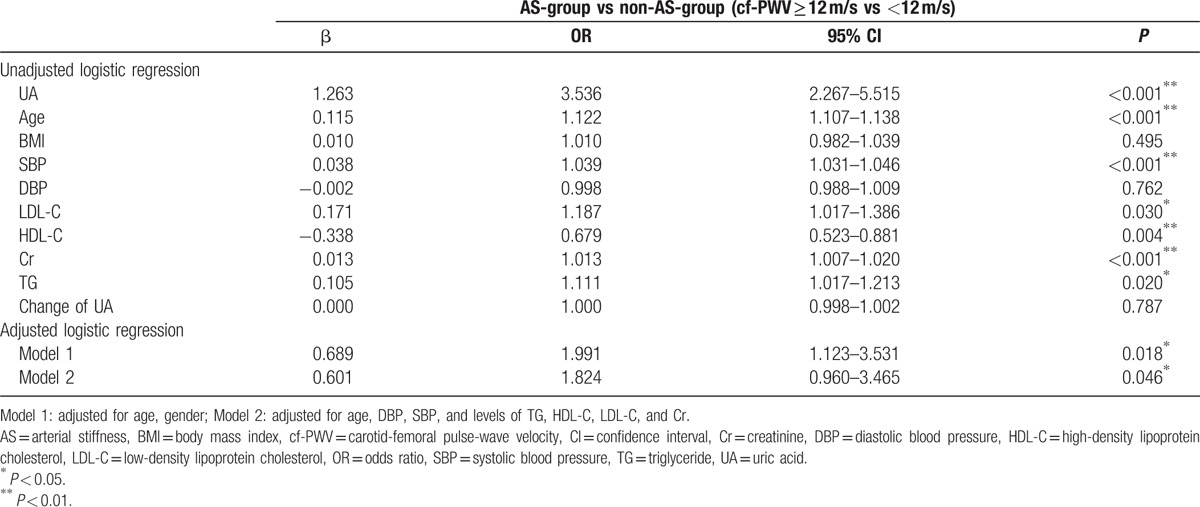
Univariate and multiple logistic regressions for baseline parameters and follow-up cf-PWV.

## 4 Discussion

In the present 4.8-year follow-up study, we found that the baseline UA level was associated with a measurement of arterial stiffness, cf-PWV. Furthermore, a higher baseline UA level was an independent predictor of follow-up central arterial stiffness (cf-PWV).

### 4.1 UA and its distribution in the health check-up population

As the end-product of purine nucleotide metabolism, UA participates in many pathophysiological processes of vascular diseases. Over the past few decades, hyperuricemia has been found to be a risk factor for cardiovascular and cerebrovascular diseases, such as atherosclerosis and hypertension.^[4,7]^ However, the predictive role of UA in health examinations is unclear.

In the present study, we observed that higher UA levels are accompanied with higher cf-PWV values, indicating that the higher range of UA may contribute to arterial stiffening. This result was partly consistent with previous studies. Furthermore, individuals with higher levels (such as Quartiles 3 and 4) of UA accounted for almost half of the health check-up population and usually presented with multiple cardiovascular risk factors. Thus, individuals with higher UA levels require proper cardiovascular risk stratification and management.

### 4.2 UA level was associated with arterial stiffness

Arterial stiffness occurs in both aortic and peripheral arteries. A large number of studies have indicated that UA is related to arterial stiffening as assessed by brachial-ankle PWV or heart-femoral PWV.^[11,24]^ In the present study, we employed cf-PWV which is a more specific measurement of central arterial stiffness, and also found an association with baseline UA.

Though previous cross-sectional studies have evaluated risk factors for arterial stiffness,^[11,24,26,27]^ the predictive value of UA level for arterial stiffness has not been confirmed. Our longitudinal survey revealed that a high UA level is an independent predictor of arterial stiffness. The present study prospectively revealed that UA correlated with aortic arterial stiffening at baseline and, in particular, a higher baseline UA was independently associated with follow-up arterial stiffness. Subclinical arterial stiffening may already exist in healthy individuals, and a high UA level may be useful in predicting the incidence of arterial stiffening prospectively.

To date, although various studies have focused on the association between UA and arterial stiffness, the mechanisms underlying the UA contribution to arterial stiffening have not been clearly elucidated. Several scientific investigations have demonstrated significant roles of UA in arterial stiffness.^[12,13,29]^

Firstly, UA contributes to the thickening of the vascular wall via promoting proliferation and differentiation of smooth muscle cells. The biological behaviors of smooth muscle cells above-mentioned are triggered by the activated renin-angiotensin system and reactive oxygen species.^[30–32]^ Secondly, studies have also indicated that the essential role of UA in arterial stiffness before the development of hypertension may be attributed to the activation of inflammatory pathways (increased levels of C-reactive protein and other proinflammatory factors).^[33]^ Thirdly, UA has also been implicated in endothelial cell dysfunction, which plays a crucial role in arterial stiffening. UA may participate in arterial stiffness via the nitric oxide pathway dysfunction, oxidative stress, and insulin resistance, which cause endothelial cell dysfunction. Endothelial cell dysfunction further leads to increased proliferation and migration of smooth muscle cells and the rearrangement of artery wall components.^[12,29]^ These changes decrease the compliance and stiffen arteries functionally and structurally.

### 4.3 Higher baseline UA level was an independent predictor of arterial stiffness

Further analysis by logistic regressions indicated that a higher baseline UA level was an independent risk factor/predictor of central stiffness. In both adjusted models, higher UA levels were associated with arterial stiffness measured by cf-PWV, indicating that a higher UA level was an independent predictor of central arterial stiffening.

Although other cross-sectional studies have identified that UA is risk for arterial stiffness, the predictive value of UA for arterial stiffness has not been confirmed in a long-term longitudinal study.^[11,24,25,34]^ Thus, we conducted this follow-up study and demonstrated that a high UA level is an independent predictor of arterial stiffness. Our observations confirm the association between UA and arterial stiffness and its predictive value for arterial stiffness.

## 5 Conclusions

A higher baseline level of UA is closely related to arterial stiffness and is an independent risk factor for and predictor of arterial stiffness. Thus, plasma UA levels may be useful for arterial stiffness risk stratification and management.

## 6 Limitations

The participants in this study were recruited from 2 districts in Beijing instead of from random sampling all over the country. The results may not be representative of Chinese individuals from other regions. The unavoidable limitation is that a total of 181 subjects (10.7%) were lost to follow-up, which may introduce bias in the conclusions.

## Supplementary Material

Supplemental Digital Content
