# Supplementary material for: A higher baseline plasma uric acid level is an independent predictor of arterial stiffness: A community-based prospective study
Source: Medicine (Baltimore). 2017 Feb 10;96(6):e5957. doi: 10.1097/MD.0000000000005957 (PMC5312993; doi:10.1097/MD.0000000000005957)
Supplement: Supplemental Digital Content [file medi-96-e5957-s001.doc]

The detailed assessments of arterial stiffness and variables definition were attached in the Appendix due to the length limitation of the manuscript.

***Appendix 1***

***Assessments of arterial stiffness in detail***

Arterial stiffness was assessed by automatic carotid-femoral PWV (cf-PWV) measurement using a Complior SP device (Createch Industrie, France) after a 5-10-min rest in the supine position in the morning, in a quiet environment, and at a stable temperature. Caffeine, smoking, and alcohol were avoided for at least 12 h before the assessment. PWV along the artery was measured with two strain-gauge transducers with a noninvasive procedure by using a TY-306 Fukuda pressure-sensitive transducer (Fukuda Denshi Co., Japan) that was fixed transcutaneously over the course of a pair of arteries separated by a known distance on the carotid and femoral arteries (both on the right side). Two transducers were used, namely, one positioned at the base of the neck over the common carotid artery and the other over the femoral artery. The measurement was repeated over 10 different cardiac cycles. PWV was calculated from the measurement of the pulse transit time and the distance traveled by the pulse between the two recording sites: PWV (m/s) = distance (m)/transit time (s). The procedure was performed by the same technicians at both the baseline and follow-up assessment.

***Definition of variables in detail***

The arterial stiffness group was defined as subjects with a cf-PWV ≥ 12 m/s. Hypertension was defined as a SBP ≥ 140 mmHg and/or a DBP ≥ 90 mmHg or the use of antihypertensive medications. DM was defined as a fasting glucose ≥ 7.0 mmol/L, glucose ≥ 11.1 mmol/L at two hours after an oral 75 g glucose challenge, or both, or use of antihyperglycemic medications. Smoking status was defined as smoking 1 or more cigarettes per day for at least 1 year. Alcohol users were defined as drinking once a week (white spirit, beer, or red wine). Body mass index (BMI) was calculated as weight (kilograms) divided by height squared (meters). At baseline, a participant was defined as having metabolic syndrome (MetS) if he or she had three or more of the following four medical conditions: (1) overweight or obesity (i.e., BMI ≥ 25.0 kg/m^2^); (2) hypertension or previously diagnosed hypertension; (3) dyslipidemia (i.e., fasting TG ≥ 1.7 mmol/L (150 mg/dL) or fasting HDL-C < 0.9 mmol/L (35 mg/dL)); (4) hyperglycemia, defined as either FPG ≥ 6.1 mmol/L (110 mg/dL), 2-h postprandial glucose ≥ 7.8 mmol/L (140 mg/dL), or a previous diagnosis of hyperglycemia.

***Appendix 2***

Table 1A. Univariate and multiple linear regression analyses at baseline

|  | **Pearson Correlation** | |  | **Multiple Linear Correlation** | | |
| --- | --- | --- | --- | --- | --- | --- |
|  | r | p |  | β | 95%CI | p |
| **cf- PWV** |  |  |  |  |  |  |
| UA^#^ | 0.183 | <0.001^**^ |  | 0.555 | -0.036~1.0146 | 0.065 |
| Age | 0.509 | <0.001^**^ |  | 0.122 | 0.101~0.143 | <0.001^**^ |
| BMI | 0.051 | 0.148 |  | 0.030 | 0.011~0.045 | 0.056 |
| SBP | 0.351 | <0.001^**^ |  | 0.044 | 0.033~0.054 | <0.001^**^ |
| DBP | 0.035 | 0.206 |  | -0.028 | -0.046~-0.010 | 0.002^**^ |
| LDL-C | 0.069 | 0.014^*^ |  | 0.161 | -0.246~0.567 | 0.438 |
| HDL-C | -0.101 | <0.001^**^ |  | 0.038 | -0.471~0.548 | 0.883 |
| Cr | 0.140 | <0.001^**^ |  | 0.026 | 0.009~0.043 | 0.003^**^ |
| TG | 0.111 | <0.001^**^ |  | 0.408 | 0.080~0.735 | 0.015^**^ |

#: natural logarithm transformed.

*: p < 0.05; **: p < 0.01.

BMI: body mass index; cf-PWV: carotid-femoral PWV; Cr: creatinine; DBP: diastolic blood pressure; FBG: fasting blood glucose; HDL-C: high-density lipoprotein cholesterol; LDL-C: low-density lipoprotein cholesterol; MetS: metabolic syndrome; PWV: pulse-wave velocity; SBP: systolic blood pressure; UA: uric acid.
